# Application of Convolutional Neural Network for Decoding of 12-Lead Electrocardiogram from a Frequency-Modulated Audio Stream (Sonified ECG)

**DOI:** 10.3390/s24061883

**Published:** 2024-03-15

**Authors:** Vessela Krasteva, Ivo Iliev, Serafim Tabakov

**Affiliations:** 1 Institute of Biophysics and Biomedical Engineering, Bulgarian Academy of Sciences, Acad. G. Bonchev Str. Bl 105, 1113 Sofia, Bulgaria; 2 Department of Electronics, Faculty of Electronic Engineering and Technologies, Technical University of Sofia, 8 Kliment Ohridski Blvd., 1000 Sofia, Bulgaria; izi@tu-sofia.bg (I.I.); tabakovsd@gmail.com (S.T.)

**Keywords:** artificial intelligence, machine learning, telemedicine, remote ECG monitoring, ECG-to-sound transformation, acoustic ECG, audio signal processing, ECG sonification

## Abstract

Research of novel biosignal modalities with application to remote patient monitoring is a subject of state-of-the-art developments. This study is focused on sonified ECG modality, which can be transmitted as an acoustic wave and received by GSM (Global System for Mobile Communications) microphones. Thus, the wireless connection between the patient module and the cloud server can be provided over an audio channel, such as a standard telephone call or audio message. Patients, especially the elderly or visually impaired, can benefit from ECG sonification because the wireless interface is readily available, facilitating the communication and transmission of secure ECG data from the patient monitoring device to the remote server. The aim of this study is to develop an AI-driven algorithm for 12-lead ECG sonification to support diagnostic reliability in the signal processing chain of the audio ECG stream. Our methods present the design of two algorithms: (1) a transformer (ECG-to-Audio) based on the frequency modulation (FM) of eight independent ECG leads in the very low frequency band (300–2700 Hz); and (2) a transformer (Audio-to-ECG) based on a four-layer 1D convolutional neural network (CNN) to decode the audio ECG stream (10 s @ 11 kHz) to the original eight-lead ECG (10 s @ 250 Hz). The CNN model is trained in unsupervised regression mode, searching for the minimum error between the transformed and original ECG signals. The results are reported using the PTB-XL 12-lead ECG database (21,837 recordings), split 50:50 for training and test. The quality of FM-modulated ECG audio is monitored by short-time Fourier transform, and examples are illustrated in this paper and supplementary audio files. The errors of the reconstructed ECG are estimated by a popular ECG diagnostic toolbox. They are substantially low in all ECG leads: amplitude error (quartile range RMSE = 3–7 μV, PRD = 2–5.2%), QRS detector (Se, PPV > 99.7%), P-QRS-T fiducial points’ time deviation (<2 ms). Low errors generalized across diverse patients and arrhythmias are a testament to the efficacy of the developments. They support 12-lead ECG sonification as a wireless interface to provide reliable data for diagnostic measurements by automated tools or medical experts.

## 1. Introduction

### 1.1. Sonification

Improving conventional diagnostic methods in clinical practice relies on the development of new artificial intelligence (AI) technologies for interpretation, risk prediction, real-time monitoring, improved noise immunity, therapy guidance, and facilitated integrations of numerous known and novel modalities for presenting physiologic signals—electrocardiogram (ECG), electromyogram (EMG), encephalogram (EEG), etc. [1,2]. AI is expected to play an increasingly important role in the diagnosis and management process as more data become available and more sophisticated algorithms are developed. A typical example of a new modality is the idea of increasing the informative value of the biosignals registered from the body surface by transforming them into sound. This hypothesis is based on the assumption that the combination of visual and auditory perception allows for a more complete interpretation of information from the surrounding environment [3]. The audio generation approach become popular under the name “sonification” and was first defined by Kramer et al. (1999) [4] as “transformation of data relations into perceived relations in an acoustic signal for the purposes of facilitating communication or interpretation”. Sonification was also defined as the process of transformation of numerical data into sound, allowing patterns in the data to be presented by auditory cues, so that humans can perceive this information through their auditory sense [5]. In recent years, the advantages of sonification were demonstrated in the auditory representation of data from various fields, such as medicine, sports, financial markets, quantum physics, meteorology, situational monitoring implementations, etc. [6,7,8,9,10,11,12]. The sonification space was defined as a useful reference system for sonification tasks [13].

Our further research covers sonification in biosignals and different medical applications, with a focus on ECG sonification: principles and implementations.

### 1.2. Sonification of EMG, EEG Signals, and Brain Scans

An example of the sonification of EMG signals was presented in [14]. Given that the EMG frequency spectrum is within the audible range, a simple solution was proposed with an audio amplifier and a loudspeaker connected at the output of an EMG recording device. The sonified EMG served as an auditory biofeedback when performing specific exercises at different loading levels, which was reported to facilitate the data interpretation by researchers. Another biofeedback rehabilitation system based on EMG sonification was designed with the purpose of aiding users in understanding the dynamic motion involving multiple muscular parts [15]. A comparison between three sonification methods that represent the EMG data in pitch, timbre, and the combination of polyphonic timbre and loudness concluded that the combined method was the most effective.

An example of using sonification to interpret brain scans was presented in [16]. The authors demonstrated a significant correlation between positron emission tomography scan images and sonification spectrograms in healthy controls vs. patients with Alzheimer’s and frontotemporal dementia. Effective detection of critical events (neonatal seizures) was achieved by listening to an audio EEG signal generated by an AI-driven sonification algorithm [17]. This study showed that an hour of EEG could be analyzed in a very short time on the order of a few seconds. Importantly, the perceptual characteristics of seizure events could be heard, yielding detection accuracy comparable to professional EEG interpreters. Another study converted three non-overlapping frequency bands (4–10 Hz, 10–20 Hz, 20–30 Hz) of the EEG spectrum to sound in a pentatonic scale (five notes per octave MIDI Octave-1) [18]. Although the results were from a case series study, this auditory representation of the EEG spectrum was found useful by 15 volunteers, who were able to distinguish five Alzheimer’s dementia patients from five healthy subjects with an accuracy of about 95%.

### 1.3. Sonification of ECG Signals

The principle behind ECG sonification lies in representing the temporal and amplitude characteristics of the ECG signal through sound. The ECG signal typically consists of waves and intervals that correspond to different phases of the cardiac cycle [19]. By mapping these features to basic auditory parameters, such as pitch, volume, and rhythm, healthcare providers or patients can listen to the ECG data and identify abnormalities or changes in the heart’s electrical activity. For the purpose of intuitive human interpretation of sonified ECG signals, two main application fields were identified [20,21]:

- Audio interpretation of abnormal heart rate values or rhythmic patterns;
- Mapping of ECG parameters for better audio representation and human perception.

In 2004, Ballora et al. [22] were one of the first to use a software sound synthesis program to create an auditory display of heart rate variability (HRV) data. The clinical application of such a method was demonstrated via playing compressed audio of long-term (Holter) ECG recordings (60 HRV events per second), giving a specific description of the HRV sound audible in four cardiac states: healthy, congestive heart failure, atrial fibrillation, and obstructive sleep apnea. For example, higher HRV in atrial fibrillation could be heard as a discontinuous jump between high and low pitches with relatively constant tinkling sounds. Further studies investigated the appropriate HRV features to improve the perceptual quality and sonification interpretability [23,24,25,26,27]. An important application was highlighted in aiding the training of athletes who were able to maintain stable target heart rates by using sonification-assisted visual monitoring of HRV [23,24]. Aldana Blanco et al. [28] presented the CardioSounds system, embedding two methods for HRV and ST-segment sonification as auditory feedback to support sports activities or monitoring ST segment changes related to myocardial infarction. Warnings of ST-segment depression above attention and alert thresholds were sonified with cascaded sounds of varying durations [29]. Further, refs. [30,31] compared different sonification methods (polarity, water ambience, stethoscope, and morph) for enhancing the audible quality of ST-segment elevation. In the conducted user studies, more than 40 participants learned to distinguish ST elevation from a normal ECG after listening to a few training examples. They showed a preference for more pleasant and natural sounds, such as water drops, over synthesized sounds commonly used in a medical context.

The extension of current ECG sonification methods was pointed out in the perspectives for mixtures of multiple channels. An example is the combination of ECG and phonocardiogram (PCG) sonifications for simultaneous auditory display of the electrical and mechanical activity of the heart [32]. For better perception and augmentation of the sounds produced by the heart’s mechanical activity, ref. [32] presented the CardioScope system for synchronous sonification of ECG and stethoscope PCG by playing them on the left and right channels, respectively. Another example is the sonification of multi-channel ECG signals for detailed auditory display of lead-specific ECG waveform alterations in different arrhythmia [33,34]. The latter concept was demonstrated by converting a six-channel ECG recording to polyphonic sounds [33,34]. The conversion technology used a note of the musical scale to give the pitch for each channel, while the channel-specific ECG amplitude modulated both volume and frequency (up to 3% variation = half of a semi-tone) to produce extended polyphonic dynamics of the audio stream. Although the results in [33] were reported in a case series study with 12 pathologic ECG recordings (6 limb leads with a duration of 10 s), this sonic-level ECG representation was found to be pleasant to listen to and useful for detecting four rhythm and conduction disturbances (atrial fibrillation, premature ventricular contraction, bigeminy, ST-elevation myocardial infarction), with 50–78% average accuracy achieved by 22 minimally trained observers. Nevertheless, the individual auditory talent was shown to be important in the ability to interpret the sonified ECG with an accuracy of up to 90%, found in almost one-third of the population. The combination of ECG sonification with ECG visualization was suggested as an option for enhanced diagnostic display with great future perspectives in clinical practice [33,34].

Studies [22,23,24,25,26,27,28,29,30,31,32,33,34] demonstrated that ECG sonification has the potential to offer real-time monitoring capabilities, enabling individuals to hear irregularities or anomalies in ECG patterns without the need for constant visual attention. Also, humans do not even have to be focused to react to sound (acoustic) alerts. However, there are fewer studies on post-processing audio ECG streams exploring the potential of AI. One approach of Camara et al. (2022) [35] converted a single-lead ECG audio stream to numerical data, representative of five musical dimensions (dynamics, rhythm, timbre, pitch, and tonality). The post-processing of these features was applied for the human identification task by means of machine learning algorithms (random forest and multi-layer perceptron). They were effectively applied for the ranking of musical features and final decision making.

Although many studies have investigated the audible sense of humans to interpret sonified ECG, improving its sonic-level representation for better perception, we find few studies focused on the automatic post-processing of sonified ECG for cardiac diagnosis, taking advantage of the growing potential of AI. The aim of this study is to develop an AI-driven algorithm for multi-channel ECG sonification, which is able to generate an acoustic stream from standard 12-lead ECG recording and then recover the original ECG data with diagnostic precision. This paper presents the original design concept of two algorithms:

- A lightweight algorithm for wearable devices that must ensure the proper generation of the acoustic stream based on the frequency modulation concept. The specific requirements are to acoustically combine the set of independent leads in standard 12-lead ECG without interaction between their data, while complying with a narrow bandwidth limited by the audio receiver.
- Deep neural network for the diagnostic server that is able to transform the acoustic stream of the sonified ECG into digital ECG signals, while maximally preserving the waveform of the original ECG at the recording site.
- This paper further describes the original ideas for the development of both modules, as well as their training and test data, with independent samples from a very large 12-lead ECG database, including more than 20,000 recordings. The final diagnostic-level information test makes use of a public biosignal processing toolbox to measure basic ECG waves and calculate the differences in amplitudes and detection times of the original vs. recovered ECG after sonification. The negligible differences found in each ECG lead are grounds for inferring the efficacy of the developments and the ability to use the recovered ECG after sonification for reliable diagnostic measurements by automated tools or medical experts.

## 2. Materials and Methods

### 2.1. Generalized Concept for Using Sonified ECG for Remote Patient Monitoring

Remote ECG monitoring generally refers to the continuous and real-time observation of a patient’s ECG data from a distance [36,37,38,39]. This form of monitoring allows healthcare professionals to assess a patient’s cardiac activity without the need for the patient to be physically present in a medical facility. The ECG signals are typically obtained through wearable devices connected to patient ECG sensors and equipped with multi-channel amplifiers for better signal quality. The data are further transmitted wirelessly to a remote location (cloud server) for analysis and interpretation. Wireless interfaces play a crucial role in facilitating the communication and transmission of reliable and secured ECG data from the patient’s monitoring device to the remote server. Various wireless technologies are commonly employed for this purpose, providing flexibility, mobility, and real-time data transfer, such as Bluetooth, Wi-Fi, cellular networks (3G, 4G, 5G), Zigbee, Internet of Things protocols, etc. [40]. The choice of wireless interface depends on factors such as the range required, power consumption, data transfer speed, and the specific use case for remote ECG monitoring. Integration with existing healthcare infrastructure and compliance with data security and privacy standards are also important considerations.

**Figure 1:**
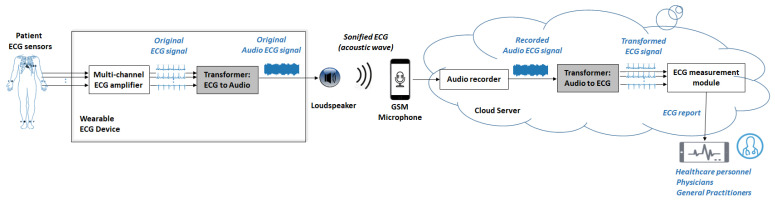
Generalized concept for using sonified ECG in remote patient monitoring.

The developments in this study are part of the general concept of using sonified ECG for remote ECG monitoring, as illustrated in Figure 1. The basic idea is that sonified ECG signals can be transmitted as an acoustic wave (over the air) and received by the microphone of a GSM (Global System for Mobile Communications) device or a tablet. Thus, the wireless connection between the patient module and the cloud server can be provided over an audio channel, such as a standard telephone call or audio message [41]. This is a procedure that does not require any special skills and can substantially help the user (patient) in self-registration and real-time sending of their ECG data to the remote end-receiver (healthcare center, physician, general practitioner, etc.). Importantly, this type of communication does not require special data protection due to the lack of RF (Radio Frequency) interfaces for short-distance data transmission. The direct use of GSM communication protocols ensures data security and privacy standards for remote communication.

Our team co-authored five of six studies we could find using a similar wireless interface that generates a sonified ECG stream and captures it with a GSM microphone [41,42,43,44,45,46], where all developments were designed for single-channel ECG processing:

- Remote recording of ECG between fingers with a commercial AliveCor device, found in a public database for screening of atrial fibrillation, including >12 k ECG recordings up to 60 s in duration, published for the PhysioNet/Computing in Cardiology Challenge 2017 [42]. Specifically, after the analogue-to-audio conversion of the ECG signal, the patient module transmitted acoustic data to a smartphone or tablet microphone, using a 19 kHz carrier frequency and a 200 Hz/mV modulation index. Software demodulation of the audio stream used sampling at 44.1 kHz and 24-bit resolution.
- Telemetry of high-risk patients with pacemakers in a laboratory study by our team [43] that reproduced the ECG recording of a patient with a cardiostimulator (down-sampled from 18 kHz to 250 Hz) in an audio stream (700 Hz carrier frequency, 100 Hz frequency deviation and amplitude modulation at pace detection instants with a duration of 200 μs). Further, the sonified ECG stream was transmitted from a PC loudspeaker to the microphone of a low-class GSM. Simple signal demodulation software sampled the audio stream at 10 kHz and was able to recover the ECG waveform and pace instants with good quality for visual recognition of heart cycles, although the ECG morphology was insufficient for precise diagnostics.
- Telemetry of high-risk patients in a cardiology unit, using a wearable ECG device equipped with a finger-based ECG sensor and ECG sonification loudspeaker, recently developed by our team [41]. In this pilot study, nine patients were successfully trained to self-record and send their sonified ECG via GSM. According to the attending cardiologist, the waveform of the remotely recovered ECGs was sufficient for monitoring of the patients’ condition.
- Fully analogue hardware solution for sonification that could convert analogue ECG signals to an audio stream using a voltage-controlled oscillator [44].
- Study of the audio characteristics of mobile phones [45] and development of a GSM modem [46] in the context of transmission of biosignals converted to sound [45].

This study is a step forward in extending ECG sonification applications from single-lead to standard 12-lead remote ECG monitoring. Our developments are further focused on the design, training, and testing of novel software algorithms for effective management of the signal processing chain of the audio ECG stream. These are the highlighted gray modules in Figure 1, including Transformer (ECG-to-Audio) and Transformer (Audio-to-ECG). The output of the second module, namely the transformed ECG signal, is sent to the ECG measurement module and the processed diagnostic information is made available for remote monitoring. Healthcare professionals can access the data, and the system may generate reports or alerts based on the analysis of the remote ECG data. Therefore, it is important that the transformed ECG signal is reliable for diagnosis, i.e., it should best reproduce the waveform of the original ECG signal acquired at the recording site.

### 2.2. Training and Testing in a PC Simulation Platform

#### 2.2.1. ECG Database and Pre-Processing

This study uses one of the largest clinical 12-lead ECG databases, namely the PhysioNet PTB-XL ECG database, version 1.0.1 [47,48]. It includes 21,837 recordings of 10 s resting 12-lead ECG, collected from 18,885 patients of whom 52% were male with median (interquartile) age of 62 (22) years. The storage resolution of the ECG data is 16-bit, 1 μV/LSB, sampled at 500 Hz. The database was originally released with the primary purpose of evaluating machine learning algorithms, making certain that the whole database is a rich representative of healthy controls and pathologic ECG rhythms (16,782 sinus rhythms, 637 sinus bradycardia, 826 sinus tachycardia, 1587 atrial fibrillations and flutters, 772 sinus arrhythmia, 157 supraventricular arrhythmia, 52 supraventricular tachycardia, 296 artificial pacemakers, and 82 bigeminia and 20 trigeminia patterns) with various diagnoses (9528 normal ECGs, 6886 myocardial infarctions, 2819 hypertrophies, 5788 ST/T changes, 5772 conduction disturbances), where each record could have one or several diagnostic labels.

The database was divided into two independent and relatively equal parts for the training (recording index 1–10,000) and testing (10,001–21,837), without exclusion or stratification of ECG pathology, amplitude, or quality.

Multiple sources of noise affect an ECG signal, such as power line interference, baseline wandering, electromyogram and motion artefacts, and electrode contact noise; therefore, filtering or other signal processing techniques should be applied for their reduction in order to provide reliable diagnostic measurements [49]. An adequate preprocessor filter design must provide maximal artifact rejection with minimal ECG distortions. However, the spectra of ECG and artifacts in their variety often overlap, implying that there would be a compromise depending on the specific application. A high-pass cut-off frequency must be selected to preserve the fidelity of the low-frequency ECG components during repolarization (ST segment), which are accurately measured in resting ECG diagnosis. Ambulatory monitoring is accompanied by strong movements, and therefore robust baseline wandering reduction must be achieved in ECG rhythm monitors, for which the recommendations are much severe, allowing a low-frequency cutoff of 0.5 Hz [50] or higher—0.67 Hz [51]. Various studies apply much higher cut-off frequencies of 1 Hz [52,53,54], 2 Hz [55], and 5 Hz [56], working under considerable artifacts of intensive body and electrode movements, such as stress testing, personal event/alarm recorders, defibrillators, etc. Such systems typically make the diagnostic interpretation based not on the ST-segment deviation, but on proper detection of the QRS complexes, rhythm analysis, or coherent PQRST template alignment. All these methods show improved accuracy when the baseline wandering is significantly rejected. For the best reduction of muscle artifacts, the low-pass cut-off frequency can vary from 40 Hz [50] down to 30 Hz [54] or 15 Hz [56].

Our study design considers first-order Butterworth filters with a cut-off frequency of 0.64 Hz (high-pass) and 30 Hz (low-pass), pursuing best artifact rejection in real-life ambulatory monitoring. This filter simulates the hardware filters that must be applied in the patient module (the multi-channel ECG amplifier in Figure 1).

#### 2.2.2. Training and Test Concept

**Figure 2:**
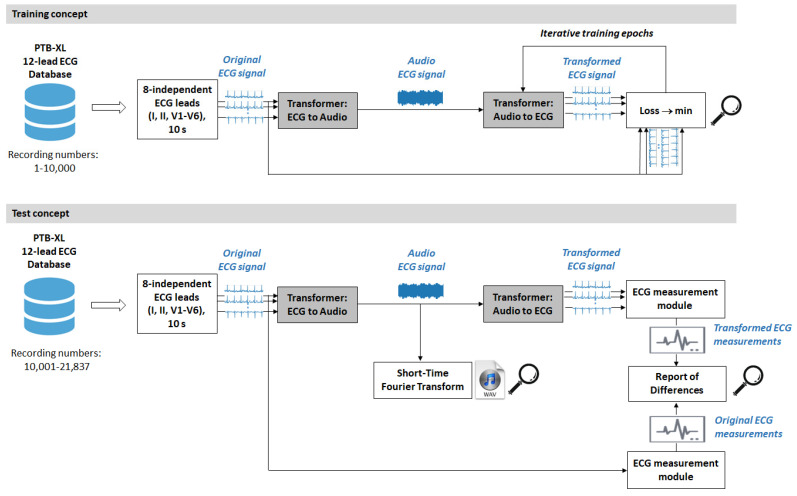
Design concept for training and testing the signal processing modules of the audio ECG stream, including Transformer (ECG-to-Audio) and Transformer (Audio-to-ECG) in a PC simulation platform. The magnifier indicates the observational points in the test process.

The diagram in Figure 2 summarizes the design of the overall process in the training and test phase of our study performed on PC.

The ECG data read from the database is limited to the minimum set of 8 independent leads (I, II, V1–V6), which are sufficient to reconstruct the 12 standard ECG leads, according to the fundamental principles of electrocardiography [57]. Ten seconds of raw data from the 8 independent ECG leads are input to the first software module under investigation, namely Transformer (ECG-to-Audio), which mixes all leads in a common audio ECG stream. The data re further fed into the second software module, Transformer (Audio-to-ECG), which outputs 10 s of 8-lead ECG, trying to reconstruct the input. This is the main signal processing chain for management of the audio ECG stream which is designed, trained, and tested in our study.

Note that the audio ECG signal in Figure 2 represents the sonified ECG in the acoustic wireless interface in Figure 1. In the PC simulation study, we do not sonify the ECG in analog format but directly use the digital audio stream in the communication path between the two transformers. We can monitor the characteristics of the audio ECG signal by supplementary analysis, including short-time Fourier transform (STFT) to analyze the frequency spectrum over 10 s, as well as recording in an audio .wav file.

The training process in machine learning performs an iterative adjustment of model parameters, which by design is applied to the Transformer (Audio to ECG). Better fit of the model to the data is obtained when the observed Loss function is minimized. In our study, Loss is defined as the root mean squared error (RMSE), which is a standard metric for measuring the average magnitude of the errors between the predicted and reference values in a regression problem. The training is unsupervised and does not involve human data annotation. It simply pursues the minimum error between the transformed and original ECG signals in all leads and sample points in the dataset (n):

(1) $$RMSE=\sqrt{\frac{1}{n}\sum_{i=1}^{n}{\left({Transformed\;ECG}_{i}-{Original\;ECG}_{i}\right)}^{2}\;}\left[\mu V\right].$$

The test phase uses the model with minimal training loss to evaluate its performance in terms of the quality of the transformed ECG signal on an independent test dataset. High-quality transformation preserves the waveform of the original ECG in the output. Therefore, the test process must estimate the total effect of the two sequential transformers (ECG-to-Audio and Audio-to-ECG) in the main signal processing chain. Generally, the quality of the transformed ECG signal is estimated by three kind of metrics:

1. Amplitude errors are represented by RMSE (Equation (1)) and the normalized RMSE to the mean ECG amplitude, namely the percentage root-mean-square difference (PRD):
  (2) $$PRD=\frac{RMS}{\frac{1}{n}\sum_{i=1}^{n}{Original\;ECG}_{i}}\times 100\left[\%\right].$$
  Although lower RMSE and PRD indicate that the transformed ECG is closer to the original ECG, their values are insufficient to assess the importance of differences for diagnostic conclusions.
2. Diagnostic errors are estimated by an ECG measurement module. Generally, the ECG diagnosis relies on measurements of specific fiducial points of basic ECG waves and intervals [57]. Ideally, if the same ECG measurement module is applied to both the original and transformed ECG signals, the measured fiducial points should coincide, i.e., give zero time offset. Therefore, the diagnostic errors are estimated as the mean absolute difference (MAD) between fiducial point times (FPTs) of the original vs. transformed ECG:
  (3) $$MAD=\frac{1}{m}\sum_{j=1}^{m}|{FPT}_{j}\left(Transformed\;ECG\right)-{FPT}_{j}\left(Original\;ECG\right)|[\mathrm{ms}],$$
  where m represents the number of fiducial points measured in a specific ECG recording and lead.

All diagnostic measurements of fiducial points and MAD were performed by means of the public open-source toolbox for biosignal processing in Python (BioSPPy, version 1.0.0) [58]. Using the embedded ECG processing routines for segmentation, including QRS detector of Christov [59] and several fiducial point detectors, the positions of the following basic ECG waves were measured in each lead: P-peak, Q-point, R-peak, S-peak, J-point, and T-peak. Additionally, the routine «compare_segmentation» was used as a tool to calculate MAD for each fiducial point and the detailed QRS detector performance (true positives (TP), false positives (FP), and false negatives (FN)). Lead-specific statistics for the total test database are reported as mean and standard deviation for MAD; Sensitivity (Se) and Positive Predictive Value (PPV) for the QRS detector, where:

(4) $$Se=\frac{\sum_{n}TP}{\sum_{n}TP+\sum_{n}FN}\times 100\left[\%\right],PPV=\frac{\sum_{n}TP}{\sum_{n}TP+\sum_{n}FP}\times 100\left[\%\right],$$

considering n as the number of ECG recordings in the test database.

3. Frequency spectrum error was computed to estimate the differences between the spectral content of the transformed and original ECGs. We defined the normalized power spectral density (PSD) error using the relation:
  (5) $$Normalized\;PSD\;error(f)=\frac{\left|{PSD}_{x}(f)-{PSD}_{y}(f)\right|}{{max(PSD}_{x},{PSD}_{y})}\times 100\left[\%\right],$$
  where:
  - x, y: the time series of the transformed ECG and original ECG signals, respectively.
  - f: the frequency at which PSD is calculated, defined in the range of the meaningful ECG spectrum (0–100 Hz).
  - PSD_x_, PSD_y_: the power spectral density of x and y, respectively. PSD is computed with Welch’s method [60,61], implemented by the routine «signal.welch» in the Python SciPy library [62].
  - max(PSD_x_, PSD_y_):The normalization factor equal to the maximum value found in the PSD of x or y signals. It is introduced to ensure a true comparison between ECG signals of different amplitudes.

### 2.3. Design of Transformer Modules

**Figure 3:**
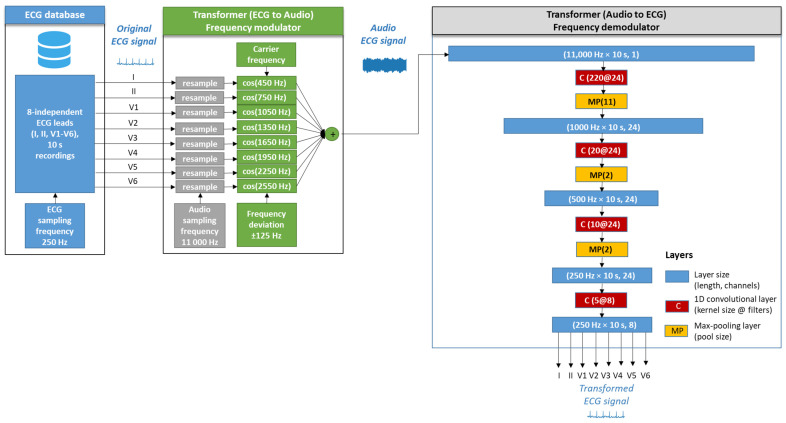
Design diagram of two modules used as a frequency modulator (FM) Transformer (ECG-to-Audio) and a FM demodulator Transformer (Audio-to-ECG).

The architecture of the two algorithms in the signal processing chain of the audio ECG stream, namely Transformer (ECG-to-Audio) and Transformer (Audio-to-ECG) are illustrated in Figure 3. Their design considerations are further communicated in detail.

#### 2.3.1. Transformer (ECG-to-Audio)

The design concept of the Transformer (ECG-to-Audio) is based on the frequency modulation (FM), which is a widely used method of encoding information in a carrier wave by varying its instantaneous frequency [63]. In the current implementation, each ECG lead is given a unique carrier frequency that is modulated by the instantaneous lead-specific ECG amplitude. Thus, the instantaneous lead-specific frequency (f) at time (t) is determined by:

(6) $$f^{L}(t)={F_{C}}^{L}+F_{D}\frac{{Original\;ECG}^{L}(t)}{A_{R}}[Hz],$$

where:

- L = 1, 2, …, 8: index of the lead taken from the original ECG lead set.
- A_R_ = 2.5 mV: the supported amplitude range of the ECG signal. All ECG amplitudes outside the range ± A_R_ are limited in the input of the transformer.
- F_C_^L^: The carrier frequency of lead L, defined as: I (450 Hz), II (750 Hz), V1 (1050 Hz), V2 (1350 Hz), V3 (1650 Hz), V4 (1950 Hz), V5 (2250 Hz), V6 (2550 Hz). Lead-specific carrier frequencies are uniformly distributed in 300 Hz steps, fitting within the limited bandwidth (300–3000 Hz) of common GSM microphones. The bandwidth is in accordance with our previous experimental study for the audio characteristics of six mobile phones of different classes [46].
- F_D_ = 125 Hz: the frequency deviation, which has a constant value for all ECG leads. The modulation index is thus defined as F_D_/A_R_ = 50 Hz/mV.
- The frequency-modulated sound signal of lead L is given by:
  (7) $$S^{L}\left(t\right)=A_{C}cos\left(\phi \left(t\right)\right)=A_{C}cos(\int_{0}^{t}2\pi f^{L}\left(t\right)dt),$$
  where A_C_ is the amplitude of the carrier frequency and ϕ(t) is the instantaneous phase, which is the integral of the instantaneous frequency over time.

Working with digital signals implies the use of the discrete form of Equations (6) and (7), where (t) is replaced by a time series index (n) using sampling frequency F_S_. Technically, this is implemented by resampling of ${Original\;ECG}^{L}(n)$ to F_S_ by means of the routine «signal.resample» in the Python SciPy library [62] that results in the discrete form of $f^{L}\left(n\right)$ in Equation (6) and $S^{L}\left(n\right)$ in Equation (7). Further, the arithmetic sum of $S^{L}\left(n\right)$ in eight ECG leads creates the digital stream of the multi-lead audio ECG signal:

(8) $$Audio\;ECG\left(n\right)=\sum_{L=1}^{8}S^{L}\left(n\right)=\sum_{L=1}^{8}A_{C}cos(2\pi \sum_{k=1}^{n}\frac{f^{L}(k)}{F_{S}}).$$

Given that the maximum modulated frequency is <3 kHz, the audio ECG data can be correctly reproduced with a sampling rate as low as F_S_ = 11 kHz. A reduced size of processed data is linked to lower computational burden on the system.

#### 2.3.2. Transformer (Audio-to-ECG)

The second processing stage in Figure 3, Transformer (Audio-to-ECG), is proposed to decode the transmitted audio ECG signal to the original 8-lead ECG data. Our design is based on a fully convolutional neural network (CNN) for FM demodulation of the ECG audio stream, which is a fairly unexplored application of this AI-driven technology. We hypothesize that convolutional filters (kernels) can be effectively self-trained to extract the useful information from FM audio signals, given that CNNs are widely used in learning hierarchical representations of input ECG signals, shown in our previous studies for arrhythmia detection [64,65]. We find literature evidence for lightweight and effective CNN applications for demodulation and automatic modulation recognition in frequency shift key, phase shift key, and quadrature amplitude modulation supported in the MHz frequency range [66,67,68]. However, we could not find studies related to CNN applications for the demodulation of very-low-band audible frequencies into ECG signals.

The detailed architecture of the designed CNN is presented in Figure 3. A CNN demodulator is applied to the audio ECG signal (duration = 10 s, sampling frequency = 11 kHz) in input layer size (length = 110,000, channels = 1) to reconstruct an 8-lead ECG signal (10 s, 250 Hz) in output layer size (2500, 8).

The CNN architecture involves four sequential 1D convolutional layers (kernel size @ number of filters = 220@24, 20@24, 10@24, 5@8) with a total number of 23,600 trainable parameters. The convolution operation is favored in signal processing tasks because it preserves the locally spatial information between sequential layers. For simplicity, assuming a single-channel input, the convolution operation can be represented as:

(9) $$Z_{i}=\alpha (\sum_{j=0}^{M-1}x_{i+j}.w_{j}+b),$$

where:

- Z_i_: the output at position (i).
- x_i+j_: the input at position (i + j).
- w_j_: the weights of the filter at position (j)—trainable parameter.
- b: the bias term—trainable parameter.
- M: the kernel size of the filter w.
- α: activation function. In this application, linear activation functions of convolutional layers are used. We note that the non-linearity of typical activation functions (i.e., rectified linear unit) is not appropriate in regression tasks, which reproduce equally ranged positive and negative magnitudes of the input to the output. In our previous study for ECG noise filtering, we showed that fully linear activation is adequate for ECG denoising and clean ECG reconstruction by convolutional autoencoders [69].

Max pooling layers are used to reduce spatial dimensions. Max pooling operation involves dividing the input into non-overlapping regions and selecting the maximum value from each region:

(10) $$P_{i}=max(p)\in pool\_region(i)X_{p},$$

where:

- P_i_: the value of the pooled output at position (i).
- X_p_: the value in the input vector at position (p).
- Pool_region(i): the region in the input vector corresponding to the pooling window centered at (i).

Three max pooling layers were used in our CNN design with respective pooling window sizes of 11 (reduces the receptive space from 110,000 down to 10,000), 2 (10,000 down to 500), and 2 (500 down to 250). These hidden layers effectively down-sample the audio stream from 11 kHz to 250 Hz, suitable for ECG sampling.

Additionally, dropout (rate = 0.1) was applied to each convolutional layer as a regularization tool to prevent overtraining on specific data by randomly setting a fraction of the neurons to zero during training.

## 3. Results

### 3.1. Implementation

Deep learning on PC requires powerful computing resources, so the experiment was conducted on a GPU-based workstation PERSY Stinger with Intel CPU Xeon Silver 4214R @ 2.4 GHz (2 processors), 96 GB RAM (Intel, Santa Clara, CA, USA), and NVIDIA RTX A5000-24 GB GPU (NVIDIA, Santa Clara, CA, USA). The system was running on Microsoft Windows Server 2019 Standard. All software modules were programmed in Python 3.9.5. Keras and Tensorflow 2.9.1 were used for the neural network implementation, training, and test.

The transformer (ECG-to-Audio) model was trained with an Adam optimizer and learning rate of 0.001. The training dataset was shuffled and fed in batches with sizes of 256 in training (70%) and validation (30%) subsets. The training process was run for a maximum of 1000 epochs; however, early stopping was activated if no improvement in the validation loss was observed for at least 150 epochs. The model with the minimum loss in the validation dataset was stored in an .hdf5 file for further test evaluation.

According to the generalized concept for using sonified ECG in remote patient monitoring (Figure 1), the audio demodulation would be implemented in the cloud server. Therefore, no special hardware constraints were applied to the server running the trained CNN model of the transformer (ECG-to-Audio). However, a lower time consumption is preferred to lighten the system and to shorten the diagnosis time. Applying statistics to all ECG recordings in the test set (>10,000), we estimated that CNN-based demodulation of eight independent ECG leads with a duration of 10 s would take 0.0506 s ± 0.0168 s (mean ± standard deviation), 0.0312–1.3710 s (min–max range). For clinical use, these times are acceptable, and even longer execution times can be tolerated in the worst-case scenario where the cloud server is not equipped with a GPU.

### 3.2. Audio ECG Signals

**Figure 4:**
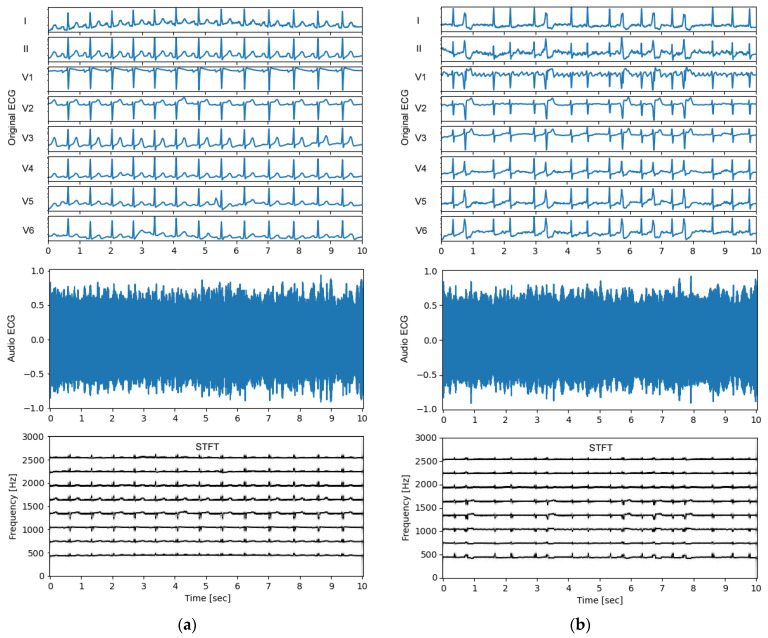
Examples of original ECG recordings (leads I, II, V1–V6) (top), generated audio ECG signals (middle) and their short-time Fourier transform (STFT) spectra (bottom) for various arrhythmias found in the PTB-XL test dataset: (**a**) Recording id 10963 from a patient with normal sinus rhythm. The ECG audio file is recorded in <Supplement S1.wav>. (**b**) Recording id 10224 from a patient with atrial fibrillation and premature ventricular contractions. The ECG audio file is recorded in <Supplement S2.wav>.

**Figure 5:**
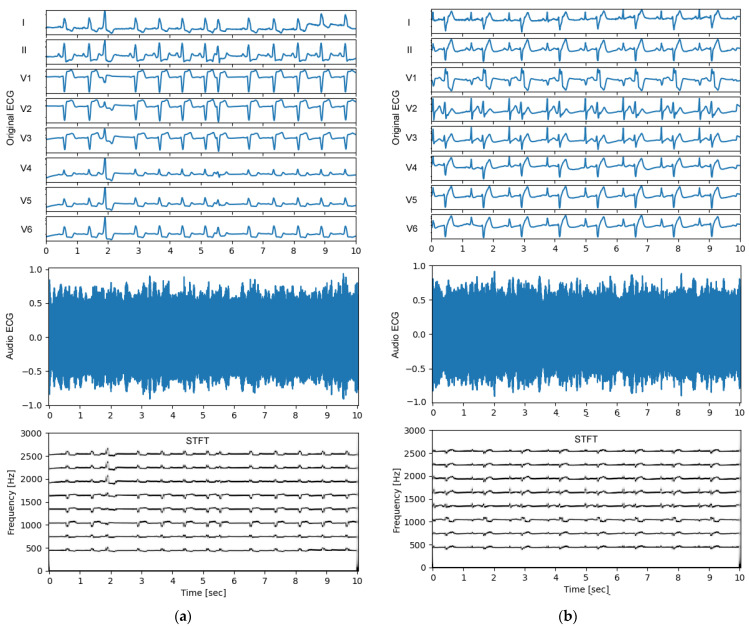
Examples of original ECG recordings (leads I, II, V1–V6) (top), generated audio ECG signals (middle) and their short-time Fourier transform (STFT) spectra (bottom) for various arrhythmias found in the PTB-XL test dataset: (**a**) Recording id 10967 from a patient with diagnostic labels for premature ventricular contraction(s), premature atrial contraction(s), sinus rhythm, left bundle branch block, and ischemic heart disease. The ECG audio file is recorded in <Supplement S3.wav>; (**b**) Recording id 10355 from a patient with diagnostic labels for premature ventricular contractions and bigeminy. The ECG audio file is recorded in <Supplement S4.wav>.

An important observational point in the test process (Figure 2, bottom) is the quality of the audio ECG signal. Feedback on the generated non-stationary audio ECG stream, and especially its frequency content over time, is obtained from the standard STFT technique, using an observation window of 1 s, shifted with a half-window overlap over the signal duration of 10 s. The results are illustrated in Figure 4 and Figure 5 for several representative examples of ECGs in patients with normal sinus rhythm and different arrhythmias. An important observation is that the ECG audio stream has a frequency content consistent with the design, i.e., eight carrier frequencies appear in STFT spectra at 450 Hz, 750 Hz, 1050 Hz, 1350 Hz, 1650 Hz, 1950 Hz, 2250 Hz, and 2550 Hz, which belong to eight ECG leads (I, II, V1–V6). The carrier frequencies change synchronously with the ECG waveform in these leads. The frequency deviation is in the range of ±125 Hz and does not support overlap between leads (frequency channels), which is an important requirement for the correct transformation of the audio to eight-lead ECG in further steps.

Additional feedback on the auditory human perception of normal sinus rhythm and various arrhythmias by the generated audio ECG streams can be obtained from the supplementary audio files: <Supplement S1.wav> (generated from the ECG rhythm in Figure 4a), <Supplement S2.wav> (Figure 4b), <Supplement S3.wav> (Figure 5a), and <Supplement S4.wav> (Figure 5b). These audio recordings are not used in diagnostic tests by human hearing. Nevertheless, they are an interesting source for analyzing the intuitive auditory perception of the audio ECG wave for immediate recognition of the heart rate and its dynamic variation in different arrhythmias, as well as pathological ECG waveform alterations.

### 3.3. Amplitude Errors

**Figure 6:**
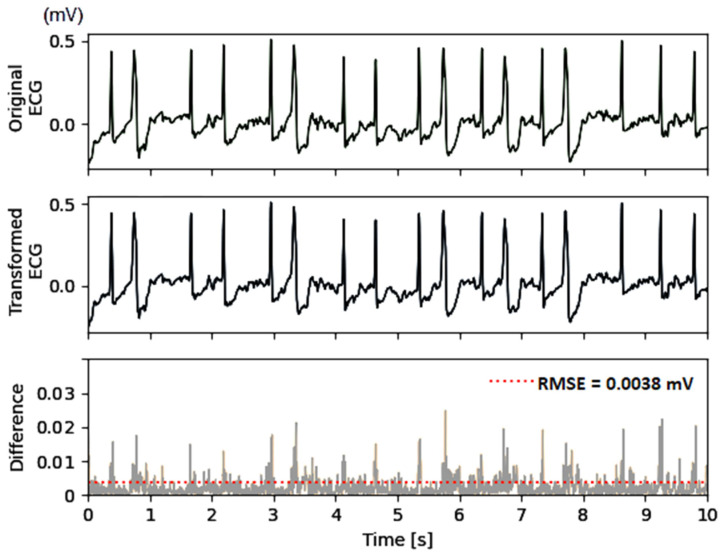
Example of original ECG (**top**), transformed ECG (**middle**), and their absolute difference (**bottom**) used for computation of the amplitude error RMSE = 3.8 μV and PRD = 3.4%. The figure reproduces the test PTB-XL dataset recording (id 10224, lead V6) of a patient with atrial fibrillation and premature ventricular contractions. The audio ECG streams of the original and transformed ECG are shown in Figure 4b.

Another important observational point in the test process (Figure 2, bottom) is the quality of the transformed ECG signal, which should ideally match the original ECG. An example of the original and transformed ECG from the test data set is illustrated in Figure 6, which shows no discernible differences between the two signals in the top and middle subplots, regardless of the notable pathological variations in the ECG waveform. High-precision computations show absolute differences with peak amplitudes of up to 30 μV, observed during the highest-amplitude QRS complexes. The error for reconstruction of small-amplitude waves (e.g., atrial fibrillatory F-waves in the example) is below 3 μV. The summary error for this specific lead is estimated using the metrics defined in equations (1,2), giving RMSE = 3.8 μV and PRD = 3.4%.

**Figure 7:**
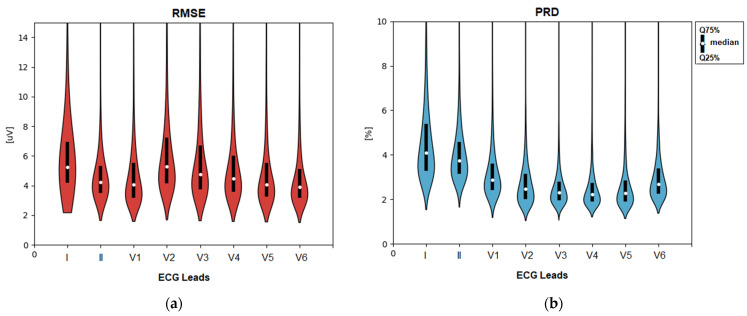
Statistical distributions of the amplitude errors measured for the transformed ECG vs. original ECG in separate leads (I, II, V1–V6) of the test set: (**a**) RMSE: root mean squared error; (**b**) PRD: percentage root-mean-square difference. The violin plot wrapping is proportional to the kernel density estimate of the underlying distribution. Median and quartile ranges are also denoted.

The statistical distributions of both errors (RMSE and PRD) calculated for the total test dataset and stratified by ECG leads are shown in Figure 7. The CNN demodulation algorithm presents substantially lower errors: RMSE of 4–5.5 μV (median), 3–7 μV (quartile range); PRD of 2.2–4.1% (median), and 2–5.2% (quartile range), where the ranges are reported as a summary of eight ECG leads. Additionally, we measured the amplitude errors in a setting with disabled monitoring-type pre-filters in Section 2.2.1, in order to investigate the influence of high-frequency ECG details and noises. These resulted in up to a 2-fold increase in amplitude errors with an RMSE of 6.2–10 μV (median), 5–14 μV (quartile range) and PRD of 4–6.5% (median), 3.2–8.5% (quartile range). It is worth noting that the latter errors represent the worst-case scenario, which is unusual in clinical practice for making diagnostic conclusions without filter control to minimize interference from noise sources.

The results in Figure 7 indicate no significant differences between the different ECG leads, giving confidence that the errors are independent of the modulating ECG amplitude and polarity, as well as the carrier frequency assigned to each ECG channel. The low errors in the independent test set are proof of the functionality of the CNN kernels in the role of self-trained FM demodulator filters.

### 3.4. Diagnostic Errors

**Figure 8:**
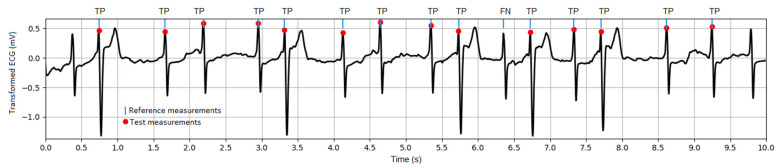
Illustration of the QRS detector performance: example of transformed ECG signal (test PTB-XL dataset, recording id 10224, lead V3) and marked R-peak positions as detected from the reference and test measurements. TP: true positives; FN: false negatives.

**Figure 9:**
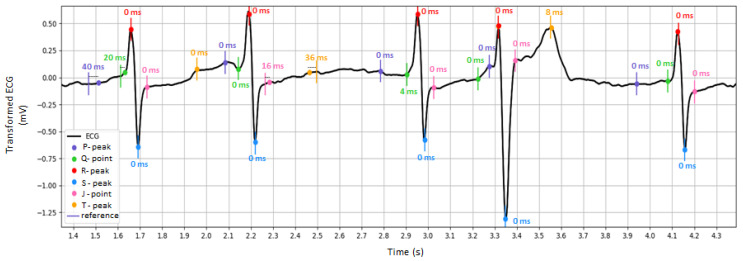
Illustration of the fiducial point measurement performance: example of transformed ECG signal (test PTB-XL dataset, recording id 10224, lead V3) and marked fiducial point positions as detected from the reference and test measurements. The absolute difference between the detection times of the reference vs. test measurements is shown next to each fiducial point.

The primary diagnostic features of the ECG waveform, including the P, QRS, and T waves, are meaningful when estimated after ECG denoising, applying the monitoring-type bandwidth defined in Section 2.2.1. Noise suppression is an important requirement to ensure reliable measurements from the ECG measurement module [58] defined in Section 2.2.2, which is not designed to operate in noisy conditions. The diagnostic errors are estimated by the ECG measurement module, which is first run on the original ECG to generate the reference measurements and then run on the transformed ECG to generate the test measurements. Generally, the difference between the test and reference measurements is considered an error. It is worth noting that each lead is processed independently by the ECG measurement module, which reports lead-specific errors. According to the methodology in Section 2.2.2, two types of performances are further reported:

1. QRS detector performance, considering a tolerance between corresponding reference and test R-peak positions equal to ±50 ms. A case example of TP and FN detections is illustrated in Figure 8. The global estimation of the QRS detector performance with a large number of heartbeats (about 135,000) in the test dataset is reported in Table 1. It shows sufficiently high Se and PPV > 99.7% in all ECG leads to conclude positively about the quality of the transformed ECG signal for correct QRS (pulse) detection.

2. Performance of fiducial point measurements, considering the absolute errors between the FPT of the test vs. reference measurements. A case example of the detected fiducial points in the transformed ECG waveform is illustrated in Figure 9. The global performance for the test dataset is reported in Table 2, deducing that the mean value and standard deviation of the fiducial point detection error does not exceed 2 ms in any lead. This is evidence of the diagnostic reliability of the transformed ECG signal.

### 3.5. Frequency Spectrum Errors

This test evaluates spectral errors over a wide frequency range relevant to ECG signals (0–100 Hz). Therefore, we disabled all preprocessing filters that were defined in Section 2.2.1. Further, the frequency error test directly uses the originally stored ECG signals in the PTB-XL database. This ensures that all ECG waveform details, including sharp deflections, rapid changes, and subtle abnormalities, as well as artifacts and noises occurring during ECG acquisition, are present in the PSD of the original and transformed ECG signals, and would be estimated by the PSD error. Such a test provides a generalized overview of the ECG spectral components beyond the frequency band of ECG monitors.

**Figure 10:**
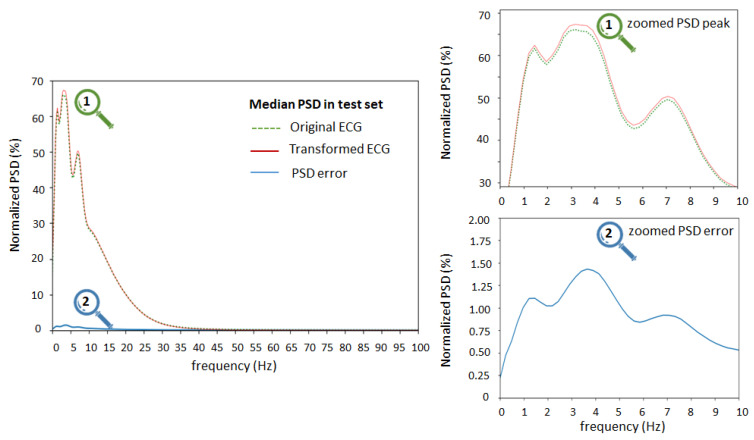
Normalized median PSD for lead II, considering original ECG, transformed ECG, and their error. On left: PSD estimation in the full frequency range 0–100 Hz. On right: zoomed zones of interest around PSD peak in the frequency range 0–10 Hz. The graphs represent the median PSD value for a specific frequency as a statistical estimate over all recordings in the test set.

PSD statistical analysis of the test set (lead II) is presented in Figure 10, showing the median value of the normalized PSD of the original ECG, transformed ECG, and their error (Equation (5)) estimated in 0.2 Hz bins from 0 to 100 Hz. The median PSD traces of original and transformed ECG considerably overlap and their difference is weakly visible. Therefore, we applied a zooming tool (on the right) to observe the zone around the PSD peak of the ECG signals in the frequency range 0 to 10 Hz. We observed slight differences between the peak spectral components of original and transformed ECG, which had the largest median values (1–1.5%) in the frequency range 1–5 Hz.

**Figure 11:**
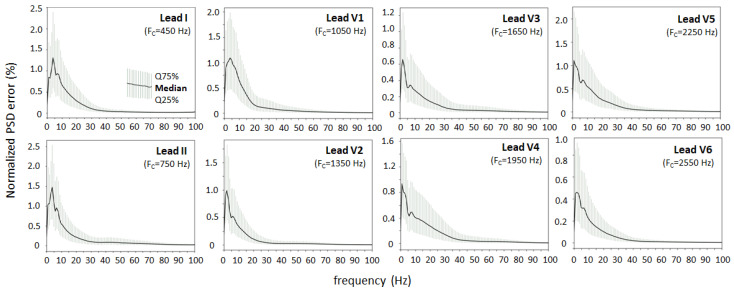
Normalized PSD error of the transformed ECG vs. original ECG, estimated in the frequency range 0–100 Hz for 8 ECG leads (I, II, V1–V6). PSD error trends are presented as median value and quartile range (Q25%, Q75%). The carrier frequency (F_C_) of each lead in the audio stream is additionally given in the legend to indicate that presented spectral errors correspond to the ECG after demodulation of specific audio frequency bands.

Figure 11 shows the normalized PSD error of the transformed ECG (Equation (5)) estimated from 0 to 100 Hz and presented separately for different ECG leads. In all leads, the peak PSD error has a similar trend to Figure 10, having a maximum in the low-frequency range 1–5 Hz, ranging from 0.5% to 1.5% (median) and 0.2% to 2.5% (quartile range). The PSD error tilt decreases with increasing frequency and drops to negligible levels < 0.1% for high frequencies > 30–40 Hz. The relatively low peak errors and similar trends of the normalized PSD error in the different ECG leads indicate that the ECG spectrum (0–100 Hz) can be equally well reproduced in the sound stream modulated at carrier frequencies from 450 Hz (lead I) to 2550 Hz (lead V6) with a frequency deviation of ±125 Hz. These differences may be caused by the combined influence of the amplitude-to-frequency conversion in the ECG-to-Audio Transformer or the CNN demodulator in the Audio-to-ECG Transformer. The hypothesis that the ECG spectrum may essentially overlap the sound frequency and therefore not be correctly interpreted in the transformed ECG signal can be rejected.

## 4. Discussion

### 4.1. Main Findings of the Study

This study presents a novel AI-driven algorithm for end-to-end transformation of a multi-lead ECG to an audio stream and its inverse transformation into diagnostic-quality ECG. The design of the algorithm is in concord with the potential clinical relevance of transforming ECG data into an audio format (ECG sonification), as outlined in Figure 1 in a scenario for remote 12-lead ECG monitoring via GSM microphones. Both patients and healthcare professionals can benefit from this transformation, as wireless interfaces are readily available, facilitating the communication and transmission of secure ECG data from the patient monitoring device to the remote server. This paper proposes a software solution to support the important problem of data reliability by answering the following questions: (1) “*How well does the algorithm process multi-lead ECG data to ensure the quality of the generated audio stream?*”, and (2) “*What is the effectiveness of the inverse transformation in reconstructing the diagnostic-quality ECG from the generated audio stream?*”.

The first question covers the design of the ECG-to-Audio Transformer, which must incorporate the principle of multiplexing to reliably transmit multiple signals (ECG leads) over the same communication channel. The methodological design for converting ECG data to sound is tailored to the limited computing resources available in the microcontroller-based portable patient modules that have the practical function of audio ECG stream generators and transmitters. We therefore decided to adapt the simple principles of classical FM modulator theory to ECG data, where both the carrier and modulation frequencies of multiple ECG channels are fit within the limited bandwidth of the GSM microphone receiver suitable for biosignals (Figure 3). Thereby, the operating frequencies are in the very low frequency range (300–2700 Hz), which favorably facilitates a reduced sampling rate to generate an audio stream at 11 kHz, saving CPU and memory usage. Such sampling frequencies are rarely used in audio recording practices, as they are qualified to give very poor sound quality. It is therefore important to provide evidence that the sonified ECG at 11 kHz preserves the essential information sufficient to reproduce the original data with diagnostic precision. This particular application is additionally complicated by the task of managing an audio ECG stream that can reproduce the diagnostic information of the standard 12-lead ECG. Due to our engineering design that seeks equal spacing between carrier frequencies of different channels (ECG leads), the frequency content of the sonified ECG is not made up of pleasant-to-hear musical tones and harmonics. The frequency spectra can be verified in the examples of normal and pathologic ECG rhythms (Figure 4 and Figure 5) with audio recordings included in supplementary. Note that they are not intended to be used for ECG diagnosis directly by human hearing. Nevertheless, our informal experience has shown that these sonified ECGs can be used for intuitive auditory detection of the heart rate and pathological variations in its dynamics in arrhythmias (e.g., atrial fibrillation and premature contractions) without special training of the listener. In addition, minimal training is required to perceive pathological changes of the ECG waveform, especially QRS widening in premature ventricular contractions and left bundle branch block beats.

The second question is related to the CNN technology embedded in the Audio-to-ECG transformer shown in Figure 3. Its effectiveness for reconstructing diagnostic-quality ECG data from an FM modulated audio stream depends on two main factors:

1. The ability of self-learning convolutional kernels to identify and respond to patterns in the input data related to the FM demodulation in unsupervised training mode, i.e., without being explicitly guided as to what those patterns are. Such feature discovery might be challenging to specify manually. In such cases, unsupervised learning is particularly useful when the characteristics of the input data are not well understood, as the model can explore and learn from statistical distributions. Therefore, the diversity of the input data is explicitly important. In this study, it is provided with 10,000 clinical ECG recordings of various pathologies.
2. The resolution of the ECG data in the FM-modulated audio stream, taking into account the design limitations of the low sampling rate (11 kHz), relatively low modulation index (50 Hz/mV), and small safety gap (50 Hz) between the highest and lowest frequencies of two adjacent FM bands. Generally, fine details in the frequency modulation, such as subtle variations or rapid changes, can be better captured with higher resolution. Beneficially, the mentioned FM resolution limitations are acceptable for the CNN demodulator, which has proven high performance for reconstructing the original ECG in a large independent test set (>11,000 clinical ECG recordings with various pathologies). Errors computed using a popular ECG diagnostic toolbox in eight independent ECG leads are substantially low: amplitude error (quartile range RMSE = 3–7 μV, PRD = 2–5.2% in Figure 7), QRS detector (Se, PPV > 99.7% in Table 1), P-QRS-T fiducial point measurements (<2 ms, Table 2). These primary diagnostic features have to be interpreted in the clinical context only if they are evaluated in the ECG monitoring bandwidth. Nevertheless, the extended overview of the ECG spectrum (0–100 Hz) in Figure 11 shows that the transformed ECG in all leads reliably reproduces the spectral components of the original ECG with a peak error up to 1.5% (median value) and up to 2.5% (upper quartile) in the low-frequency range (1–5 Hz), and <0.1% in the high-frequency range (>30–40 Hz). Nevertheless, we cannot directly link the observed errors of the median spectrums to meaningful clinical measurements of ECG amplitudes. These errors can be due to the audio modulator and demodulator. Generally, there is no evidence of difficulties in reconstructing the original ECG spectrum from the audio stream, although the carrier frequencies of different leads deviate from 450 Hz (lead I) to 2550 Hz (lead V6). Obviously, our FM design does not meet problems related to potentially harmful overlap between the ECG and audio spectra. All the aforementioned tests were performed with a large ECG test set, giving statistical evidence of the diagnostic reliability of the transformed ECG signal in all leads and generalization across diverse patients and arrhythmias.

To the best of our knowledge, this is the first study to investigate the CNN applications for FM demodulation of very-low-band audible frequencies, solving the regression problem for recovery of ECG signals. Its concept is different than the classic FM demodulators, the most popular of which use frequency-selective carrier networks with an envelope detector, slope detector, phase-shift detector, ratio detector, quadrature detector, etc.; the pulse averaging discriminator method, including a zero-crossing detector, monoshot multivibrator and low-pass filter; or the PLLs (Phase-Locked Loops) circuit with phase-locked integrated circuits or voltage-controlled oscillator to generate a reference signal of frequency equal to the carrier frequency [70]. Notably, classic FM demodulators use several connected modules that need specialized engineering design and settings, implying that they may require handcrafted features and fine-tuning for different signal characteristics; may need labeled data for training; are less adaptable to complex or varying signal conditions; and show increasing complexity with signal bandwidth. Unsupervised deep learning is easier and more suitable for scenarios with varying or complex signal characteristics. Although the training can be computationally intensive, the interference of a model based on convolutional operations is not computationally demanding and, if necessary, can be accelerated with hardware accelerators (GPUs, TPUs) in the cloud.

### 4.2. Contemporary Techniques for Remote ECG Monitoring

Remote patient monitoring systems have been widely used for decades in telemedicine as they play a vital role in remote cardiac monitoring, enabling timely diagnosis, intervention, and management of cardiac disease, even in geographically dispersed or resource-constrained settings [71]. Conventional ECG monitoring systems have evolved to address various challenges in accuracy, efficiency, and practical applicability, driven by advances in technology, validation processes, and clinical acceptance [38,72,73].

The accuracy issues are majorly related to signal quality, given that the accuracy of remote ECG monitoring systems heavily relies on the quality of the ECG signals captured and transmitted from the patient to the monitoring center. Advancements in sensor technology [74,75,76,77,78,79,80,81,82], signal processing algorithms [83,84], and noise reduction techniques [81,85,86] have improved signal quality, leading to more accurate ECG interpretations. The more leads used, the more noise and false positive alarms emerge, and therefore ECG monitoring is usually limited to one- to three-lead settings with unconventional electrode placement [74,84,85,86,87,88]. Nevertheless, 12-lead ECG is the clinical standard for cardiac diagnosis based on detailed ECG waveform segmentation and rule-based cardiologist interpretations. Therefore, some studies applied advanced techniques for increasing the amount of informative leads, such as reconstruction of some standard leads and asynchronous ECG acquisition from several anatomic positions [77,87,89]; and the use of advanced wearable devices with multiple sensing electrodes embedded in chest rings or patches [75,76] and fabric electrodes in T-shirts [79,80]. Recent studies demonstrate 12-lead ECG systems for home use with facilitated and individually deformable placement of the sensors [76,81]. Bench tests and clinical validation of wearable ECG sensor systems typically check for compliance with the standard 12-lead ECG based on common diagnostic metrics, such as:

- QRS detection accuracy of about 96.6% in rest [84] down to 85% during jogging [81] with special measures provided to improve noise immunity.
- Fiducial point measurement of P, Q, R, S, T with errors for laying (2.4 ± 0.5 ms), sitting (0.6 ± 10.9 ms), and walking (6.6 ± 14.7 ms) persons [80].
- Interval duration errors differ between leads and studies and are reported with approximate maximal ranges: RR interval (50 ± 100 ms), QRS duration (1 ± 15 ms), QT interval (3 ± 30 ms), PQ interval (10 ± 50 ms) [75,88,89].
- PRD is reported to be about 5.2% [82].

In terms of accuracy, our method provides at least the same and even better performance of the transformed ECG measurements. However, we should take into account that our results were generated from a simulation study and they may deviate in a clinical setting. It is worth noting that this method can use input data from different kinds of ECG sensors connected to the patient module, considering that the generated audio stream can transfer from one to eight ECG leads without the need for adaptation of the transmission protocol or data content.

The efficiency covers real-time monitoring capabilities, allowing healthcare providers to receive and review ECG data promptly. This is the major feature of this and other ECG monitoring systems, despite the disparities between the efficiency of the data transmission protocols. A common approach in various ambulatory systems is to transmit data over a short distance from the patient module to a mobile (GSM) or fixed device on the Internet (Gateway), and then relay it to a remote end receiver. Low-power interfaces are used for close communication (e.g., Bluetooth Low Energy, Zigbee, WiFi) [74,82,90,91]. Problems with such implementations are related to establishing the connection, maintaining the communication channel, and packet losses. Even at home, if the distance between the transmitter and receiver is large, or there are fences or other sources of signals in the same frequency range, communication may not be possible [92]. Furthermore, Bluetooth has some security issues while data are transferring [93].

Integration with healthcare systems such as telemedicine platforms enhances the practical applicability of remote ECG monitoring solutions. This requires transferring and storing big data in the virtual space (cloud) through long-distance communication by modern technologies, such as the IoT, 4G, and 5G, providing connectivity everywhere. Their essential advantages are the relatively easy WPS (Wireless Protected Setup) procedure for joining the network; the availability of a standardized communication protocol MQTT (Message Queue Telemetry Transport) for data transmission to the cloud; different levels of QoS (Quality of Service) system communication setting; and encrypted SSL/TLS protocols [94]. It should be noted that the built-in network module providing IoT connectivity has a high current consumption of about 30–40 mA in reception mode and up to 100–220 mA in transmission, which largely limits the application of this type of communication in mobile sensor systems with autonomous power [95].

Conventional remote ECG monitoring systems can benefit from user-friendly interfaces that are easy to use for both patients and healthcare providers. Intuitive design and navigation enhance practical applicability by minimizing training requirements and user errors. The idea for the conceptual implementation described in this article emerged after analyzing the problems in patient telemetry, and in particular the use of new technologies for data transfer. Such problems have been found in some of the largest target groups—the elderly and chronic disease patients, who have the highest incidence of cardiovascular diseases. A number of studies [73,91,96,97,98] related to the implementation of new information and communication technologies in remote patient monitoring have shown that many of these people may have difficulties in trusting, accepting, and using new technologies, mainly due to lack of previous exposure or experience with the technology; difficulties in adapting to changes; resistance to learning new skills; and difficulty using small screens, buttons, and navigating digital interfaces. Similar problems are observed in people with visual impairments. They face difficulties in accessing and using remote monitoring devices mainly due to lack of visual information and tactile cues [99,100,101]. Incorporating familiar elements, such as physical buttons or analog interfaces (audio in this case), can make new technologies more acceptable for them. In summary, according to the Deloitte Center for Healthcare report, remote patient monitoring not only faces technological challenges from sensors and protocols but also social, cultural, and educational challenges from patients and their convictions [91].

Referring to the above information on the performance and practical applicability of remote ECG monitoring, we can summarize the following pros and cons of the proposed ECG sonification interface for short-range communication:

- Pros: The transmission of the sonified ECG signal between the patient module and the nearby GSM is performed without collisions related to lack of connectivity and packet losses; limited possibility for data hacking; does not require the development of user applications (apps) for GSM; uses standard voice communication and all benefits of 4G and 5G protocols and connectivity; easy handling, as data transmission takes place only by dialing the telephone number of the final recipient (doctor, medical server); two-way communication allowing instruction of the patient during ECG recording; low consumption of the audio generator, comparable to Bluetooth Low Energy.
- Cons: The transmission of the sonified ECG signal must be carried out in the absence of strong ambient noise; when connecting multiple users, a telephone exchange must be used.

### 4.3. Future Works Related to Clinical Implementation of Sonified ECG

Although this version of the algorithm for ECG sonification has not yet undergone implementation and validation in real-world clinical settings, such a step should comply with the following important considerations:

- Diverse patient populations and clinical contexts—The use of a large ECG database in the simulation phase of training and testing is the first important requirement to replicate real world conditions as closely as possible. Optional retraining or testing with other relevant databases with different cardiac pathologies and/or noises recorded in test benches or clinical settings are typical best practices to prepare the machine learning algorithms for clinical tests.
- Continuous monitoring, evaluation and improvement—The performance of the algorithm must be checked regularly based on clinical records and optional improvements can be considered by incorporating feedback from healthcare professionals, new data from specific clinical cases, and emerging best practices.
- Ethical considerations regarding patient privacy, data security, and compliance with regulatory frameworks governing medical devices and AI-driven diagnostics—In order to use AI-driven technology, the necessary approvals and certifications for medical devices and clinical research plans must be obtained from regulatory authorities. Telemetric sonified ECG devices (single-channel) are currently being used in a single-center clinical trial for diagnostics and monitoring of heart rhythm and conduction disorders under the supervision of Bulgarian regulatory authorities (Ethical Commission for Clinical Investigations ECCI Ref. 4469/06.10.2023 for EUDAMED), in accordance with Regulation (EU) 2017/745 of the European Union on the clinical investigation and sale of medical devices for human use, including good clinical practice, and informed consent and data management practices providing security, privacy, and confidentiality of personal data. An additional advantage related to ensuring data security can be related to the transmission of a sonified ECG stream at very close distances (<20 cm from the patient module to the GSM), which cannot be directly hacked, unlike wireless interfaces operating in the RF or long-distance ranges.
- Scalability of the proposed solution in respect to challenges that might arise in integrating it into existing healthcare infrastructure, especially in resource-constrained settings—Our current experience of using the sonified ECG interface with GSM connectivity in clinical setting is still limited, based on two preclinical studies with volunteers [41,43] and an ongoing clinical trial in the Cardiology Clinic of the Medical University—Sofia (approval disclosed in previous paragraph), including 50 consecutive patients with rhythm and conduction disturbances. The obtained results demonstrate the possibility of rapid integration into an existing hospital infrastructure, providing the monitoring and diagnostic process. First, it is not necessary to engage additional personnel for continuous monitoring of incoming data. Second, patients report that they find the “event recorder” mode of operation convenient and easy to use, where the patient independently records their own ECG and sends it as a voice message to the medical server. No hardware or connectivity issues are reported to delay the prompt sending of the data. In the diagnostic site, the cardiologist receives a message on a mobile device about the incoming record, and can later download and analyze it at their convenience.
- Implement measures to enhance user experience, both for healthcare professionals interpreting sonified ECG signals and for patients interacting with the system—Patient education and engagement is important, first to ensure a quality ECG audio stream and second to empower patients to actively participate in their care by explaining the significance of ECG signals, interpreting sonifications, and recognizing warning signs or abnormalities. Training can provide reassurance to patients using the system. The patient feedback questionnaire on the convenience of using portable sonified ECG devices in GSM telemetry is important to make improvements and address concerns. Comprehensive training and support must be provided to healthcare personnel on the use of sonified ECG devices, especially in settings where technical expertise may be limited. User-friendly interfaces and educational materials can be developed to facilitate adoption and proficiency among healthcare providers. By implementing different measures, healthcare professionals and patients can benefit from an enhanced user experience with the sonified ECG system, leading to improved clinical outcomes, increased efficiency, and greater satisfaction with the healthcare delivery process.
- The algorithm must ensure data fidelity and integrity during the transformation of ECG signals into audio streams—The sonified ECG stream is generated by the portable telemetry module, and therefore its hardware must be powerful enough to perform digital FM modulation. The FM-modulated audio stream in this study is simulated with considerations for minimal resource requirements at low audio resolution (16-bit) and limited sampling rate (11 kHz). In all cases, portable system design and laboratory testing should be planned to check for any potential problems related to FM audio signal degradation due to hardware limitations. Such laboratory tests for the GSM receiver have already been conducted to verify the audio characteristics of mobile phones [45] and the development of a GSM modem [46] in the context of transmission of biosignals converted to sound [45].

## 5. Limitations of the Study

This is a simulation study in which the audio stream from a sonified multi-lead ECG is transferred directly as digital data between an ECG-to-Audio Transformer and Audio-to-ECG Transformer. We were unable to investigate the distortions that would occur as a result of environmental and noise effects on this audio stream, as well as speaker and microphone nonlinearities in real wireless communications. In general, noise immunity can be increased if a GSM with built-in ambient noise cancellation is used. This would be an interesting subject for future research.

It is worth noting a limitation in the real-life use of sonified ECG as an acoustic interface, related to the short distances that must be maintained between the patient module and the GSM microphone (<20 cm in previous experimental studies [41,43,44,45,46]). Nevertheless, keeping such short distances is not difficult to ensure, given that the patient is in close contact during routine GSM use. Since these are negligible distances, we probably would not face common problems in the long-distance transmission of FM signals, where components of the composite spectrum could be differently attenuated.

## 6. Conclusions

The methods in this study supervise a novel short-range audio interface for standard 12-lead remote ECG monitoring with GSM connectivity to the cloud server. The original contribution is a front-end technology for generating and decoding the audio stream, containing a sonified multi-lead ECG. The first innovation is related to the synchronous superposition of eight ECG leads in a single FM audio stream with a very low frequency band (300–2700 Hz), compatible with GSM microphones. The second innovation is related to the decoding of the FM audio stream by an AI-driven algorithm. The useful information extracted from the FM audio is the raw data of an eight-lead ECG that should best reproduce the input ECG from the patient. Applying machine learning and quantitative analysis with >20,000 ECG recordings from a rich arrhythmia database, the methods are validated to transform an audio stream into eight independent ECG leads (I, II, V1–V6) with low amplitude errors (quartile range: RMSE = 3–7 μV, PRD = 2–5.2%), sufficient diagnostic quality (QRS detector: Se, PPV > 99.7%, P-QRS-T fiducial points’ time deviation < 2 ms), and acceptable error of the frequency spectrum (quartile range: 0.2–2. 5% for 0–100 Hz). Low errors generalized across diverse patients and arrhythmias are a testament to the efficacy of the developments. They support 12-lead ECG sonification as a wireless interface in remote ECG monitoring, providing reliable data for diagnostic measurements by automated tools or medical experts.

## Figures and Tables

**Table 1:** Performance of the QRS detector run on the transformed ECG signals of the test dataset.

QRS Detector	I	II	V1	V2	V3	V4	V5	V6
TP	134,597	133,722	131,962	134,269	134,577	135,014	135,173	135,004
FP	134	190	370	189	111	84	107	138
FN	181	179	363	170	141	92	141	98
Se, %	99.87	99.87	99.73	99.87	99.90	99.93	99.90	99.93
PPV, %	99.90	99.86	99.72	99.86	99.92	99.94	99.92	99.90

**Table 2:** Mean absolute difference (MAD) between fiducial point times of original vs. transformed ECG estimated in the test dataset. Results are reported as mean value ± standard deviation.

MAD	I	II	V1	V2	V3	V4	V5	V6
P-peak, ms	0.5 ± 1.1	1.5 ± 1.7	1.0 ± 1.6	1.1 ± 1.7	1.3 ± 1.8	1.3 ± 1.8	1.1 ± 1.7	1.2 ± 1.7
Q-point, ms	0.5 ± 1.0	0.9 ± 1.5	0.9 ± 1.5	0.8 ± 1.3	0.7 ± 1.3	0.8 ± 1.4	0.9 ± 1.5	1.0 ± 1.6
R-peak, ms	0.2 ± 0.6	0.3 ± 0.6	0.4 ± 0.9	0.2 ± 0.6	0.2 ± 0.6	0.2 ± 0.4	0.1 ± 0.4	0.2 ± 0.5
S-peak, ms	0.4 ± 0.8	0.3 ± 0.7	0.1 ± 0.4	0.2 ± 0.5	0.3 ± 0.6	0.3 ± 0.6	0.3 ± 0.5	0.4 ± 0.6
J-point, ms	0.6 ± 1.2	1.2 ± 1.6	1.0 ± 1.6	0.7 ± 1.2	0.7 ± 1.2	0.7 ± 1.3	1.0 ± 1.5	1.3 ± 1.7
T-peak, ms	0.3 ± 0.9	0.8 ± 1.4	0.5 ± 1.0	0.5 ± 1.0	0.6 ± 1.2	0.8 ± 1.5	0.9 ± 1.6	1.0 ± 1.6

## Data Availability

The PTB-XL dataset is publicly available through the PhysioNet website at https://physionet.org/content/ptb-xl/1.0.1/, last accessed on 15 January 2024.

## Supplementary Materials

The following supporting information can be downloaded at: https://www.mdpi.com/article/10.3390/s24061883/s1, This paper includes audio recordings of ECG streams with frequency modulation of eight independent ECG leads (I, II, V1, V2, V3, V4, V5, and V6) at frequencies of 450 Hz, 750 Hz, 1050 Hz, 1350 Hz, 1650 Hz, 1950 Hz, 2250 Hz, and 2550 Hz, respectively. The original ECG signals are taken from the standard 12-lead ECG in the PTB-XL database [47,48] and converted to audio ECG recordings: <Supplement S1.wav> (audio ECG from a patient with normal sinus rhythm in Figure 4a, PTB-XL ecg_id 10963); <Supplement S2.wav> (audio ECG from a patient with atrial fibrillation and premature ventricular contractions in Figure 4b, PTB-XL ecg_id 10224); <Supplement S3.wav> (audio ECG from a patient with premature ventricular contractions, premature atrial contractions, sinus rhythm, left bundle branch block, and ischemic heart disease in Figure 5a, PTB-XL ecg_id 10967); <Supplement S4.wav> (audio ECG from a patient with premature ventricular contractions and bigeminy in Figure 5b, PTB-XL ecg_id 10355).
